# Inhibitory effects of monoterpenes on human TRPA1 and the structural basis of their activity

**DOI:** 10.1007/s12576-013-0289-0

**Published:** 2013-10-12

**Authors:** Masayuki Takaishi, Kunitoshi Uchida, Fumitaka Fujita, Makoto Tominaga

**Affiliations:** 1 Division of Cell Signaling, Okazaki Institute for Integrative Bioscience, National Institute for Physiological Sciences, National Institutes of Natural Sciences, Higashiyama 5-1, Myodaiji, Okazaki, Aichi 444-8787 Japan; 2 Central Research Laboratories, Mandom Corporation, Osaka, 540-8530 Japan; 3 Department of Physiological Sciences, The Graduate University for Advanced Studies, Okazaki, 444-8585 Japan

**Keywords:** Monoterpene, Pain relief, TRPA1, Hydroxyl group

## Abstract

TRPA1, one of the transient receptor potential channels, has been reported to be involved in nociception and inflammatory pain, suggesting that this molecule could be a promising target for the development of analgesic agents. We screened several monoterpene analogs of camphor, which is known to inhibit human (h) TRPA1, to identify more effective naturally occurring TRPA1 antagonists. Borneol, 2-methylisoborneol, and fenchyl alcohol exhibited higher inhibitory effects on hTRPA1 activity than either camphor or 1,8-cineole. Our results revealed further that the S873, T874, and Y812 residues of hTRPA1 were involved in the inhibitory effects, suggesting that the hydroxyl group in the six-membered ring of the inhibitors may be interacting with these amino acids. Further research on these identified TRPA1 antagonists could lead to new pain therapeutics.

**Electronic supplementary material:**

The online version of this article (doi:10.1007/s12576-013-0289-0) contains supplementary material, which is available to authorized users.

## Introduction

Transient receptor potential (TRP) channels respond to a wide variety of sensory stimuli, including temperature, nociceptive compounds, touch, osmolarity, and pheromones [1–3]. TRPA1, one of the TRP channels, functions as a receptor that responds to noxious cold temperatures and pungent compounds, including allyl isothiocyanate (AITC), a component of mustard oil [4–8]. Although the role of TRPA1 in sensing noxious cold stimulus and somatic mechanosensation in vivo remains unsettled, especially in mammals [5, 6, 9], TRPA1 has been established as a chemical nocisensor for a wide variety of reactive compounds, such as flufenamic acid (FFA), 2-aminoethoxydiphenyl borate, icilin, menthol, intracellular calcium, and zinc ions [7, 10–18]. In addition, in previous studies we identified TRPA1 as a receptor for irritation of the skin induced by parabens [19] and for pain produced by alkaline pH [20]. In contrast, menthol has different effects on TRPA1 in humans and mice. Several compounds, such as menthol, have been found to have a bimodal action on mouse TRPA1 (mTRPA1) gating, with submicromolar to low micromolar concentrations of menthol causing robust channel activation and higher concentrations leading to reversible channel blocking [21, 22]. Such bimodal action was not observed with human TRPA1 (hTRPA1) [21]. TRPA1 has also been reported to be involved in inflammatory processes, including inflammation produced by several airway irritants that cause asthma [23–25] and neuropathic pain [23]. Therefore, TRPA1 is an excitatory ion channel targeted by acute nociception and inflammatory pain and is considered to be a promising target for the development of analgesic agents [26–31].

Previous studies showed that the inhibitory effects of AMG5445, a compound partially activating mTRPA1 and inhibiting hTRPA1 [28], and of AP18, a compound that inhibits hTRPA1 [9], can be nullified by mutations at serine and threonine located in the transmembrane domain 5 (TM5) of hTRPA1 [21]. CMP1, a close analog of AMG5445, contributes to channel blocking at a serine and isoleucine located in TM6 of hTRPA1 [32]. Monoterpenes, such as menthol, camphor, and 1,8-cineole, comprise a group of naturally occurring organic compounds derived from essential oils that have been used for anesthetic, analgesic [31, 33, 34], anti-inflammatory [31, 35], and antipruritic applications [36, 37]. As these compounds share similarities in terms of chemical structure, it is not surprising that they interact with the same molecular target for their analgesic effect [38]. Menthol has been shown to be an activator of mTRPA1 at low concentrations and a blocker at high concentrations [21, 22]. Xiao et al. [21] showed that at high concentrations of menthol, serine 876 and threonine 877 of mTRPA1 contributed to the inhibitory effects on mTRPA1 activation, suggesting that threonine 877 might form a hydrogen bond with menthol. Camphor and 1,8-cineole, both well-known components of essential oils, are reported to exert analgesic effects through the inhibition of TRPA1 [31, 39] and activation of TRPM8 [39, 40]. However, the structural basis of hTRPA1 inhibition by these two compounds remains unclear.

Several TRPA1 antagonists have been reported: 1,8-cineole contained in eucalyptus oil [39], camphor obtained from the* Cinnamomum camphora* tree [31], HC-030031 [27], AZ868 [41], A-967079 [42], and CMP1, CMP2, and CMP3 (the latter three identified as thioaminal-containing molecules [32]). Among these TRPA1 antagonists, naturally occurring analgesic compounds that inhibit hTRPA1 and which have demonstrated a safety profile based on long usage would be desirable. Indeed, we recently reported that 1,8-cineole is a rare natural compound that both inhibits hTRPA1 and activates hTRPM8 [39]. Several compounds with similar structures exhibit different effects on hTRPA1. For example, menthol and 1,4-cineole activate hTRPA1, while camphor and 1,8-cineole inhibit hTRPA1 [39]. Given these promiscuous effects on hTRPA1, more detailed analyses would lead to a better understanding of the structural basis for the action of these compounds with TRPA1 [39].

We screened camphor analogs to identify more effective TRPA1 antagonists. From this screening, we found that borneol, 2-methylisoborneol, and fenchyl alcohol exhibited higher inhibitory effects than camphor and 1,8-cineole. In addition, we found that the S873, T874, and Y812 residues of TRPA1 were critically involved in the inhibitory effect of borneol.

## Materials and methods

### Molecular cloning

Full-length hTRPA1 was obtained from Life Technologies (Carlsbad, CA). cDNAs were cloned into the pcDNA3.1 vector.

### Reagents

Camphor, borneol, fenchyl alcohol, and 2-methylisoborneol were obtained from Wako Pure Chemical Industries Ltd. (Osaka, Japan). (−)-Fenchone, 1,8-cineole, camphorquinone, norcamphor, α,β-thujone, α-pinene oxide, (−)-limonene oxide, (+)-borneol, (−)-borneol, and (±)-isobornyl methyl ether were obtained from Sigma-Aldrich (St. Louis, MO). Bornyl acetate, (±)-isoborneol, and 3-methylene-2-norbornanone were obtained from Tokyo Kasei Co. Ltd. (Tokyo, Japan). The compounds were used as a mixture of (+) and (−) isomers unless otherwise stated.

### Cell culture

Human embryonic kidney (HEK) 293T cells were maintained in DMEM (WAKO Pure Chemical Industries Ltd.) supplemented with 10 % fetal bovine serum (Biowest SAS, Caille, France), 100 U/mL penicillin (Life Technologies), 100 μg/mL streptomycin (Life Technologies), and 2 mM l-glutamine (GlutaMAX; Life Technologies) at 37 °C in 5 % CO_2_. For Ca^2+^-imaging, 1 μg of plasmid DNA containing hTRPA1 in pcDNA3 in OPTI-MEM medium (Life Technologies) was transfected into HEK293T cells using Lipofectamine Plus Reagent (Life Technologies). Following incubation for 3–4 h, cells were reseeded on coverslips and incubated further at 37 °C in 5 % CO_2_.

### Ca^2+^-imaging

Ca^2+^-imaging was performed 1 day after transfection. HEK293T cells on coverslips were mounted in an open chamber and superfused with a standard bath solution (140 mM NaCl, 5 mM KCl, 2 mM MgCl_2_, 2 mM CaCl_2_, 10 mM HEPES, and 10 mM glucose, pH 7.4). Cytosolic-free Ca^2+^ concentrations in HEK293T cells were measured by dual-wavelength fura-2 (Molecular Probes, Invitrogen Corp.) microfluorometry with excitation at 340/380 nm and emission at 510 nm. The fura-2 ratio image was calculated and acquired using the IP-Lab imaging processing system (Scanalytics Inc, Fairfax, VA). Ionomycin was used to confirm cell viability in the vector-transfected cells.

### Electrophysiology

Whole-cell patch-clamp recordings were performed 1 day after transfection. The standard bath solution was the same as that used in the Ca^2+^-imaging experiments, and extracellular Ca^2+^ was removed and 5 mM EGTA added for the recording of AITC-, menthol- and FFA-induced current responses. The pipette solution contained 140 mM KCl, 5 mM EGTA, and 10 mM HEPES, pH 7.4 (adjusted with KOH). Data from the whole-cell voltage-clamp recordings were sampled at 10 kHz and filtered at 5 kHz for analysis (Axon 200B amplifier with pCLAMP software; Axon Instruments, Sunnyvale, CA). The membrane potential was clamped at −60 mV for all conditions. In some experiments, voltage ramp-pulses from −100 to +100 mV (500 ms) were applied every 5 s. All experiments were performed at room temperature.

### Data analysis

Data in all of the figures are shown as the mean ± standard error of the mean, and *P* values of <0.05 were considered to be significant. Statistical significance of the effects of borneol, 1,8-cineole, and camphor on hTRPA1 mutants were evaluated using Student’s *t* test. Dose-dependent curves were fit with a Hill equation.

## Results

### Screening of naturally occurring compounds having effects on hTRPA1

**Fig. 1:**
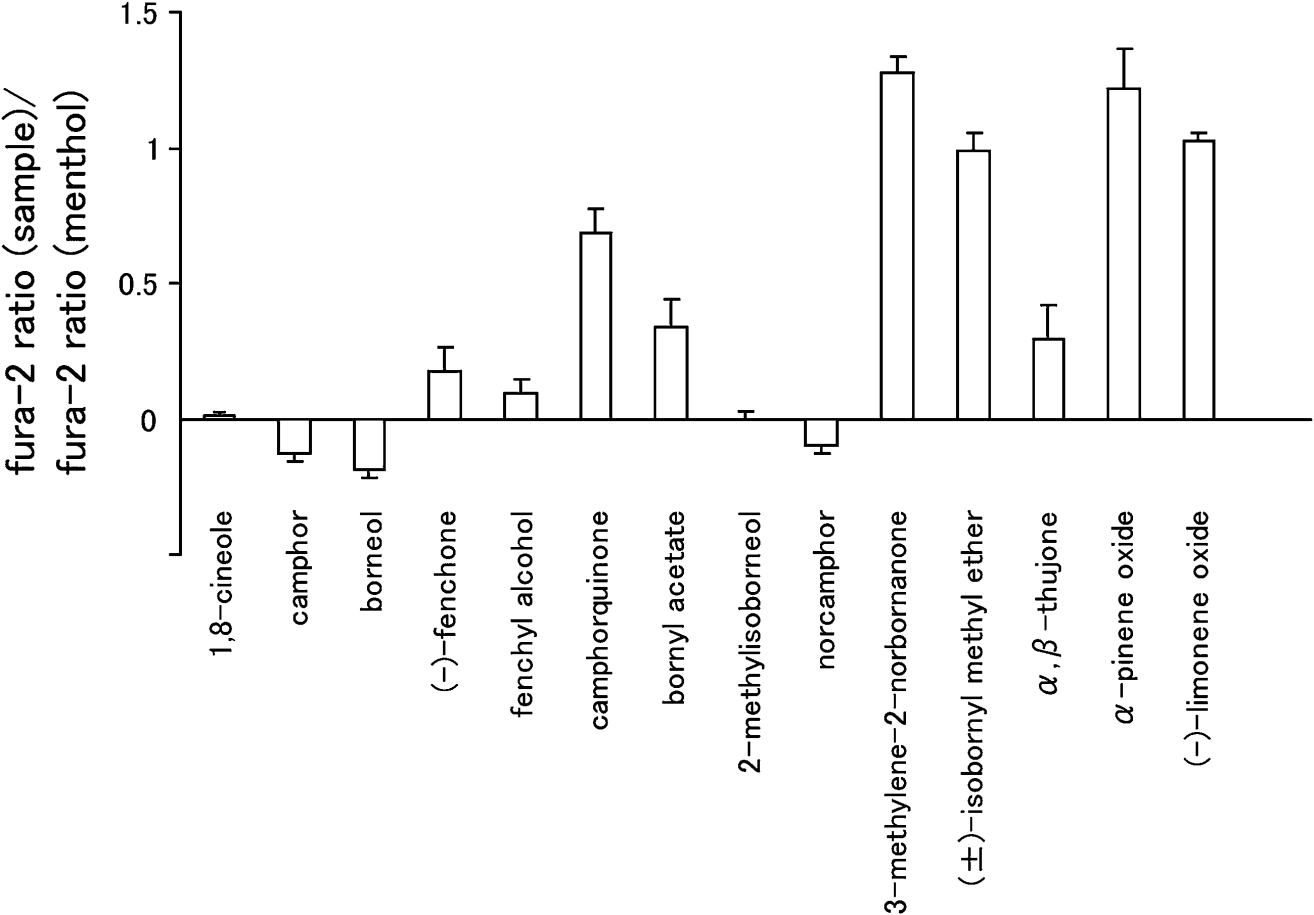
Summary of the inhibitory effects of camphor analogs on the activity of human transient receptor potential channel TRPA1 (hTRPA1) activity. Fura-2 ratios (340/380 nm) by test compounds (1 mM) were normalized to changes in the fura-2 ratio by 1 mM menthol in human embryonic kidney (HEK) 293T cells expressing hTRPA1. Data are presented as the mean ± standard error of the mean (SEM) (*n* = 27–67)

Because camphor is known to inhibit hTRPA1, we first examined the effects of camphor analogs, many of which are present in essential oils (Table 1), on hTRPA1 using a Ca^2+^-imaging method with hTRPA1-expressing HEK293T cells. In these experiments, changes in the fura-2 ratio (corresponding to cytosolic Ca^2+^ concentrations) induced by the test compounds and menthol were compared because menthol, which activates hTRPA1, and the test compounds are members of the monoterpene family. Borneol, 2-methylisoborneol, norcamphor, and fenchyl alcohol showed small changes in the fura-2 ratio, similar to 1,8-cineole and camphor (Fig. 1), which suggests that these compounds do not activate hTRPA1.

**Table 1:** Chemical structures of camphor analogs and menthol^a^

Compound	Structure
1,8-Cineole	
Camphor	
Borneol	
(−)-Fenchone	
Fenchyl alcohol	
Camphorquinone	
Bornyl acetate	
2-Methylisoborneol	
Norcamphor	
3-Methylene-2-norbornanone	
(±)-Isobornyl methyl ether	
α,β-Thujone	
α-Pinene oxide	
(−)-Limonene oxide	

^a^Hydroxyl, carbonyl, and ether oxygen are indicated in red

### Effects of borneol, 2-methylisoborneol, fenchyl alcohol, and norcamphor on hTRPA1-mediated current responses

**Fig. 2:**
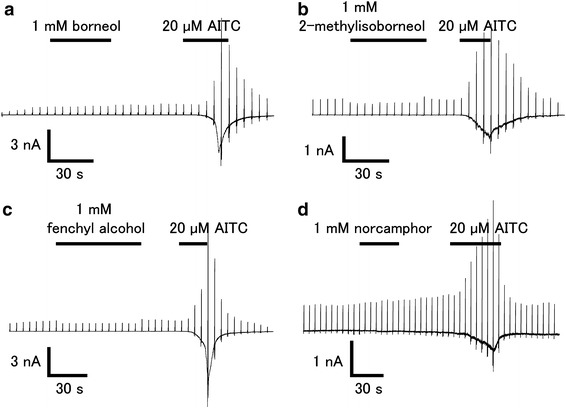
Effects of borneol, 2-methylisoborneol, and fenchyl alcohol on HEK293T cells expressing hTRPA1. Representative whole-cell current traces in the presence of borneol (1 mM, **a**), 2-methylisoborneol (1 mM, **b**), fenchyl alcohol (1 mM, **c**) or norcamphor (1 mM, **d**) in HEK293T cells expressing hTRPA1. hTRPA1 activity was confirmed with 20 μM of allyl isothiocyanate (*AITC*). Cells were held at −60 mV and ramp-pulses from −100 to +100 mV (500 ms) were administered every 5 s

In order to confirm the effects of the above compounds, we performed patch-clamp experiments with HEK293T cells expressing hTRPA1. As shown in Fig. 2, borneol, 2-methylisoborneol, fenchyl alcohol, and norcamphor did not activate hTRPA1, while AITC evoked a robust current activation with outward rectification.

### Borneol, 2-methylisoborneol, and fenchyl alcohol, but not norcamphor inhibit hTRPA1 in a Ca^2+^-imaging method

**Fig. 3:**
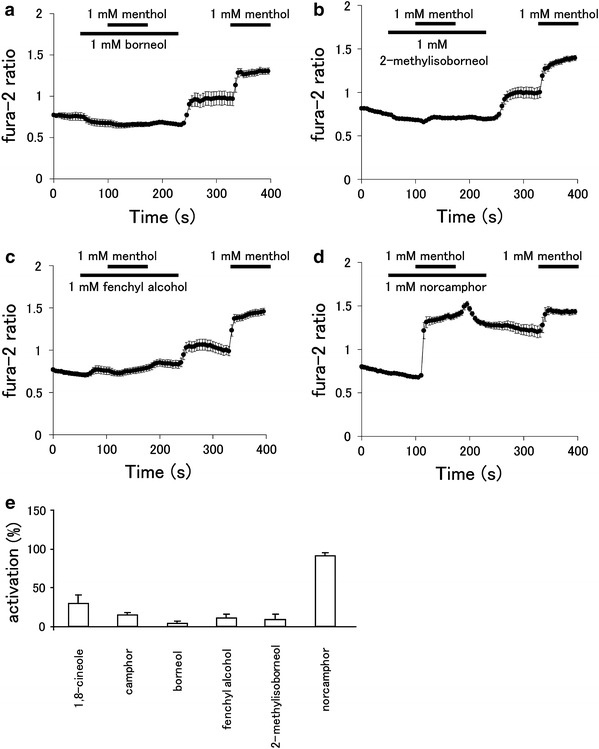
Effects of borneol, 2-methylisoborneol, fenchyl alcohol, and norcamphor on menthol-induced cytosolic Ca^2+^ increases in HEK293T cells expressing hTRPA1. Fura-2 ratio changes due to menthol (1 mM) application in the presence and absence of borneol (1 mM, **a**), 2-methylisoborneol (1 mM, **b**), fenchyl alcohol, (1 mM, **c**), and norcamphor (1 mM, **d**) in cells expressing hTRPA1 (*n* = 14–27). **e** Changes in fura-2 ratios due to menthol in the presence of test compounds were normalized to changes in the fura-2 ratio by menthol in the absence of test compounds. Data are presented as the mean ± SEM (*n* = 47–79)

We next investigated the possibility that the above four compounds inhibit hTRPA1 using a Ca^2+^-imaging method with hTRPA1-expression HEK293T cells. Increases in the fura-2 ratio caused by menthol were almost completely blocked in the presence of borneol (1 mM), 2-methylisoborneol (1 mM), and fenchyl alcohol (1 mM) in a similar manner as 1,8-cineole and camphor (Fig. 3a–c, e). Washing-out of the three compounds after menthol exposure led to small increases in the fura-2 ratio, which could be due to the loss of inhibition and resumption of hTRPA1 activity from residual menthol in the cell. In contrast, norcamphor did not inhibit the menthol-induced increase in the fura-2 ratio (Fig. 3d, e). We confirmed that borneol, 2-methylisoborneol, fenchyl alcohol, and norcamphor did not provide any effect on vector-transfected cells, while cells responded normally to ionomycin (5 μM) [Electronic Supplementary Material (ESM) Fig. 1]. These results suggest that borneol, 2-methylisoborneol, and fenchyl alcohol, but not norcamphor, inhibit hTRPA1 activity.

### Borneol, 2-methylisoborneol, and fenchyl alcohol inhibit hTRPA1 current in a dose-dependent manner

**Fig. 4:**
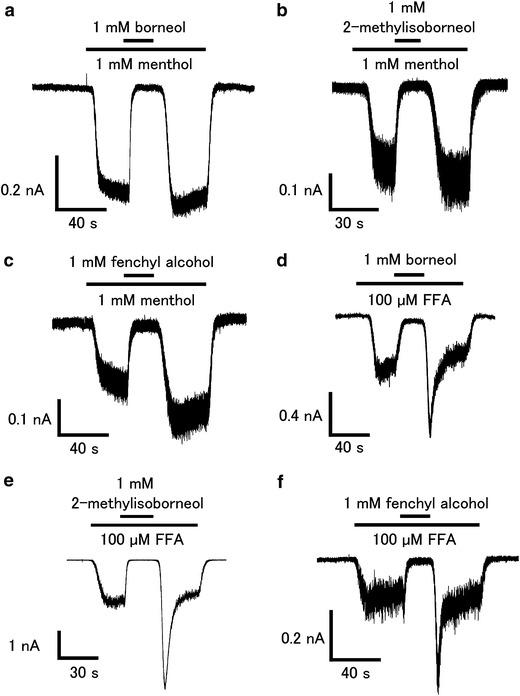
Effects of borneol, 2-methylisoborneol, and fenchyl alcohol on menthol- and flufenamic acid (FFA)-induced hTRPA1 currents in HEK293T cells.** a**–**c** Representative menthol (1 mM)-induced hTRPA1 current that was inhibited by borneol (1 mM, **a**), 2-methylisoborneol (1 mM, **b**), or fenchyl alcohol (1 mM, **c**) in the absence of extracellular Ca^2+^.** d**–**f** Representative FFA (100 μM)-induced hTRPA1 current that was inhibited by borneol (1 mM, **d**), 2-methylisoborneol (1 mM, **e**), or fenchyl alcohol (1 mM, **f**) in the absence of extracellular Ca^2+^

**Fig. 5:**
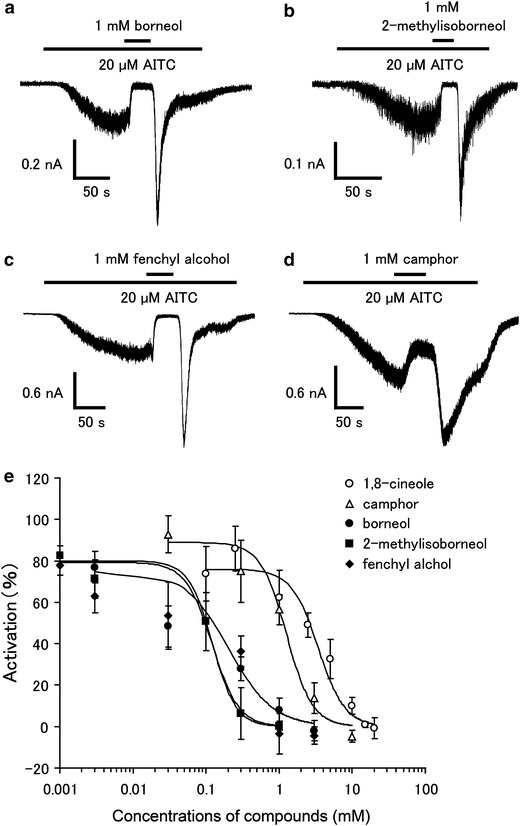
Effects of borneol, 2-methylisoborneol, fenchyl alcohol, and camphor on AITC-induced hTRPA1 current in HEK293T cells.** a**–**d** Representative AITC (20 μM)-induced hTRPA1 currents that were inhibited by borneol (1 mM, **a**), 2-methylisoborneol (1 mM, **c**), fenchyl alcohol (1 mM, **c**), or camphor (1 mM, **d**) in the absence of extracellular Ca^2+^. **e** Dose-dependent inhibition of AITC (20 μM)-induced hTRPA1 current by 1,8 cineole, camphor, borneol, 2-methyl isoborneol, or fenchyl alcohol. Half-maximal inhibitory concentrations (IC_50_) values are 3.43 ± 0.58, 1.26 ± 0.32, 0.20 ± 0.06, 0.12 ± 0.03, and 0.32 ± 0.06 mM for 1,8-cineole, camphor, borneol, 2-methylisoborneol, and fenchyl alcohol, respectively (*n* = 5–10)

To confirm the inhibition of hTRPA1 activity by the three compounds, we performed patch-clamp experiments with HEK293T cells expressing hTRPA1. The current response was measured in the absence of extracellular Ca^2+^ to minimize desensitization. 1 mM of borneol, 2-methylisoborneol, and fenchyl alcohol completely inhibited the hTRPA1-mediated current activated by menthol (1 mM) or FFA (100 μM) (Fig. 4). Enhancement of the hTRPA1 current was observed upon washing-out of the three compounds in response to activation by FFA, but not in response to that by menthol. We then determined the effective concentrations of the three compounds for the inhibition of hTRPA1. For this experiment, AITC was chosen as the hTRPA1 agonist because this molecule has a higher ability to activate hTRPA1 than menthol or FFA. 1 mM of borneol, 2-methylisoborneol, and fenchyl alcohol completely inhibited the AITC (20 μM)-induced hTRPA1 current, while 1 mM camphor partially inhibited the AITC-induced response (Fig. 5a–d), suggesting that borneol, 2-methylisoborneol, and fenchyl alcohol are more able to inhibit hTRPA1 than camphor. Enhancement of the hTRPA1-current was again observed upon washing-out of the three compounds. The dose-dependency of the inhibitory effects of the three compounds on hTRPA1 was then examined using the patch-clamp method. Similar to camphor and 1,8-cineole, the hTRPA1 current induced by AITC (20 μM) was inhibited by borneol, 2-methylisoborneol, and fenchyl alcohol in a dose-dependent manner, with half-maximal inhibitory concentrations (IC_50_) of 0.20 ± 0.06, 0.12 ± 0.03, and 0.32 ± 0.06 mM, respectively, which are much lower than the IC_50_ of 1,8-cineole (3.43 ± 0.58 mM) and camphor (1.26 ± 0.32 mM) (Fig. 5e). These data suggest that borneol, 2-methylisoborneol and fenchyl alcohol have the potential to be effective analgesic compounds. Commercially available borneol contains both optical isomers. We confirmed that there was no difference in the inhibitory effects on hTRPA1 activity between (+)- and (−)-borneols (ESM Fig. 2).

### The hydroxyl group of borneol contributes to inhibition of human TRPA1

**Fig. 6:**
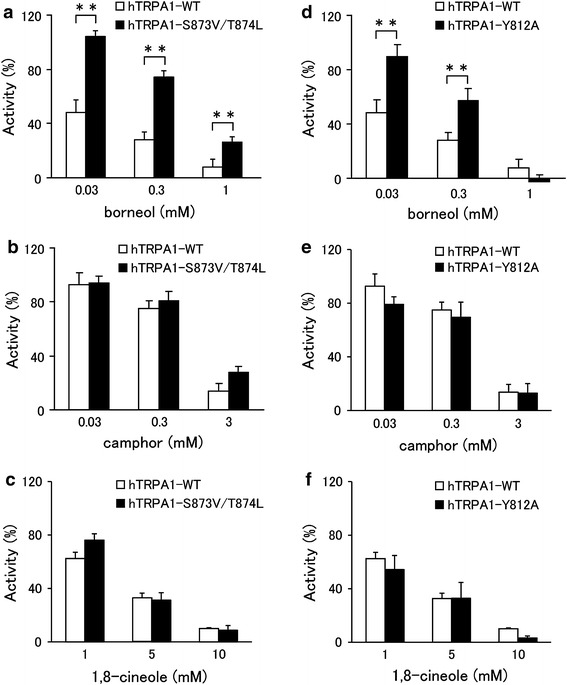
Comparison of the inhibitory effects of borneol, camphor, and 1,8-cineole on the currents of wild-type hTRPA1 (*hTRPA1-WT*) and hTRPA1 mutants (*hTRPA1-S873V/T874L*,* hTRPA1-Y812A*) expressed in HEK293T cells. Inhibitory effects of borneol at three different concentrations (0.03, 0.3, and 1 mM, **a**), camphor at three different concentrations (0.03, 0.3, and 3 mM, **b**), or 1,8-cineole at three different concentrations (1, 5, and 10 mM, **c**) on TRPA1-mediated current at −60 mV in HEK293T cells expressing hTRPA1-WT or TRPA1 mutants hTRPA1-S873V/T874L (**a**–**c**) or hTRPA1-Y812A (**d**–**f**), in the absence of extracellular Ca^2+^. Current magnitude in the presence of borneol, camphor, or 1,8-cineole was normalized to the current in the absence of test compounds. (*n* = 5–8) **P < 0.01

Xiao et al. [21] showed that menthol acts as an activator of mTRPA1 at low concentrations and as a blocker at high concentrations. This bimodal effect of menthol on TRPA1 was observed in a mouse clone, but not in a human clone. A mouse TRPA1 mutant in which serine and threonine residues located in the predicted inner side of TM5 were replaced with valine and leucine (S876V/T877L), respectively, was neither activated nor inhibited by menthol [21]. In this same study, the serine and threonine residues were found to be critical for determining the sensitivity of TRPA1 to menthol in both mammalian TRPA1 channels. These findings led the authors to suggest that T877 of mTRPA1 interacts with menthol through a hydrogen bond [21]. The fact that borneol, 2-methylisoborneol and fenchyl alcohol have a similar hydroxyl group in their structures led us to hypothesize that these compounds interact with the same serine and/or threonine of TRPA1 in a similar manner as menthol. To test this hypothesis, we investigated whether the serine and threonine residues are involved in the inhibitory effect of borneol. The inhibitory effects of borneol on the mutant channel (hTRPA1-S873V/T874L, corresponding to mTRPA1-S876V/T877L) were significantly lower than those on the hTRPA1-WT, based on the effects on the AITC-activated hTRPA1 current at three different concentrations (Fig. 6a). On the other hand, camphor and 1,8-cineole showed no significant changes in their inhibitory effects on cells expressing mutant hTRPA1 compared with hTRPA1-WT (Fig. 6b, c). We searched for an involvement of other amino acids in borneol activity. Because a tyrosine residue in TM2 of TRPM8 is known to be involved in interactions with menthol, we screened tyrosine mutants in TM2 and TM3 of hTRPA1 using a Ca^2+^-imaging method and found that the effect of borneol was reduced with the Y812A mutant (data not shown). In terms of the effect on the AITC-activated hTRPA1 current, results from the patch-clamp studies revealed that borneol had a significantly lower inhibitory effect on the mutant (hTRPA1-Y812A) at two different concentrations than on hTRPA1-WT, whereas such differences between WT hTRPA1 and hTRPA1-Y812A were not observed for 1,8-cineole or camphor activity (Fig. 6d–f).

## Discussion

In this study, camphor analogs were screened to identify more effective naturally occurring TRPA1 antagonists and to clarify the structural basis of their respective activity. Borneol, 2-methylisoborneol, and fenchyl alcohol were identified as more potent antagonists of hTRPA1 than camphor and 1,8-cineole. Moreover, the S873, T874, and Y812 residues were found to be critically involved in the activity of borneol with hTRPA1, likely through an interaction with its hydroxyl group.

TRPA1 is an excitatory ion channel targeted by pungent irritants, such as those from mustard oil and garlic, and is thought to function in diverse sensory processes, including nociception and inflammatory pain. As such, TRPA1 is a promising target for the development of analgesic agents. Although several TRPA1 antagonists, such as ruthenium red, HC-030031, AMG5445, A967079, and camphor, have been reported to possess analgesic properties [26–31], naturally occurring analgesic compounds which inhibit hTRPA1 would be desirable because of their demonstrated safety during long-term usage. Of the known hTRPA1 antagonists, camphor is a naturally occurring compound that is often used in cosmetics because of its minimal adverse effects. We recently found that 1,8-cineole is another naturally occurring hTRPA1 antagonist. However, these naturally occurring antagonists exhibit weaker inhibitory effects on TRPA1 activity than other antagonists, such as HC-030031, A-967079, and AZ868. Therefore, the identification of naturally occurring compounds with a greater potency for inhibiting hTRPA1 activation is eagerly awaited.

Borneol, a bicyclic monoterpenoid alcohol that has been used in foods as an aromatic spice, is a valuable medical and chemical material that has been used as a folk medicine in China and India [43]. Additionally, borneol is a fragrance ingredient used in decorative cosmetics, fine fragrances, shampoos, and other toiletries. Previous studies have shown that borneol has a vasorelaxant effect on the rat thoracic aorta [44] and neuroprotective effects [45]. Although borneol has been evaluated for anti-nociceptive and anti-inflammatory activities, the molecular targets and mechanisms of its analgesic effect remain unclear. The fact that this monoterpene acts as an agonist of the TRPV3 [40] and TRPV1 [31] channels and specifically inhibits nicotinic acetylcholine receptor (nAChR)-mediated effects in a noncompetitive way [46] does not explain its anti-nociceptive effects. 2-Methylisoborneol has been implicated as a cause of the muddy odor of fish from Cendar Lake, Manitoba. Algae produce 2-methylisoborneol and geosmin, which are responsible for the musty odor [47]. Fenchyl alcohol, which is a component of several essential oils, is a fragrance ingredient used in decorative cosmetics, fine fragrances, shampoos, and other toiletries. This compound also has an inhibitory effect on acetylcholinesterase activity [48]. Although borneol is the only terpene known to have anti-nociceptive effects, the two other terpenes identified in this study could have similar effects since all three monoterpenes inhibit hTRPA1; consequently, their derivatives might function as analgesics.

Alpizar et al. [49] recently reported that camphor exhibits a bimodal effect on mTRPA1. These authors observed the inhibition of mTRPA1-mediated basal current with 1 mM camphor, with an increase in the current upon washing-out of camphor, suggesting that camphor had a bimodal action on mTRPA1. In addition, 1–5 mM camphor increased the cytosolic Ca^2+^ concentration upon washing-out without any effect during camphor application. We examined the camphor (1 mM) effect in a Ca^2+^-imaging method, but failed to observe changes in the cytosolic Ca^2+^ concentrations (Fig. 1). We did observe blockage of the menthol (1 mM)-induced increase in cytosolic Ca^2+^ concentrations by camphor (1 mM, Fig. 3e) and also observed that camphor inhibited the AITC (20 μM)-activated TRPA1 current in a dose-dependent manner (Fig. 5e). At this time, we are unable to provide an explanation for the apparent differences between the two studies, although a species difference might have caused the different outcomes. Transient enhancement of TRPA1 current was observed after washing-out of the three test compounds in FFA- or AITC-evoked responses (Figs. 4, 5), but not in the menthol-evoked response (Fig. 4). The bimodal actions of several compounds on mammalian TRPA1 has mainly been reported for mammalian TRPA1 agonists involving non-covalent mechanisms. However, Alpizar et al. [49] reported that cinnamaldehyde (a TRPA1 activator with covalent modification) and camphor, which was thought to be a mammalian TRPA1 antagonist, also exhibit bimodal actions on mTRPA1, indicating that bimodal actions could be a more general phenomenon than previously thought. Therefore, the transient enhancement of the human TRPA1 current upon washing-out of the identified antagonists observed in our study might also result from a bimodal action of the compounds. Interestingly, enhancement was not observed in the menthol-evoked TRPA1 response (Fig. 4), possibly because menthol and hTRPA1 inhibitors (borneol, 2-methylisoborneol, and fenchyl alcohol) are all monoterpenes that act at similar sites, including ones which were identified in this study, through their cyclohexyl hydroxyl groups.

Analogs of camphor exhibited promiscuous effects on hTRPA1. Borneol, 2-methylisoborneol and fenchyl alcohol inhibited hTRPA1, while norcamphor had no effect on hTRPA1, and other related compounds activated hTRPA1 (Fig. 1). The mechanisms of action with hTRPA1 remain unclear, although these monoterpenes have similar molecular structures. The inhibitory effects of hTRPA1 by borneol, which was synthesized by the chemical reduction of camphor, were greater than those of camphor. In addition, fenchyl alcohol, which was synthesized by the chemical reduction of fenchone, inhibited hTRPA1 activity, while fenchone activated hTRPA1. Common structural differences between camphor and borneol and between fenchone and fenchyl alcohol are hydroxyl and carbonyl groups at the same position of their six-membered rings, which suggests that hydrogen bonding plays a pivotal role in the action of these compounds. This notion is supported by the observations on the mutation of T874, an amino acid thought to form a hydrogen bond with menthol; this mutation results in reduced activity of borneol. In our study, S873 and T874 in TM5 and Y812 in TM3 were found to be involved in the inhibitory effects of borneol. Because these two sites are somewhat distant from each other, borneol could fit separately into both sites.

In conclusion, we screened monoterpenes to identify more effective naturally occurring TRPA1 antagonists and found that borneol, 2-methylisoborneol, and fenchyl alcohol exhibited higher inhibitory effects on hTRPA1 than camphor and 1,8-cineole. Moreover, three amino acids of hTRPA1, namely, S873, T874 and Y812, were found to be involved in the activity of borneol. Further research on borneol, 2-methylisoborneol, and fenchyl alcohol could lead to the development of anti-nociceptive agents through TRPA1 inhibition.

## Electronic supplementary material

Below is the link to the electronic supplementary material.

Supplementary material 1 (JPEG 324 kb) **Supplementary Figure 1. Effects of borneol, 2-methylisoborneol, fenchyl alcohol and norcamphor with or without menthol on cytosolic Ca** ^**2+**^ **concentrations in vector-transfected HEK293T cells.** No changes in the fura-2 ratio were observed while cells responded normally to ionomycin (5 μM). (n = 48-75)

Supplementary material 2 (JPEG 126 kb) **Supplementary Figure 2** Comparison of the inhibitory effects on AITC (20 μM)-induced hTRPA1 current among borneol isomers and (±) isoborneol. (n = 5-6)

## References

[1] Christensen AP, Corey DP (2007). TRP channels in mechanosensation: direct or indirect activation?. Nat Rev Neurosci.

[2] Minke B, Cook B (2002). TRP channel proteins and signal transduction. Physiol Rev.

[3] Zhang XF, Chen J, Faltynek CR, Moreland RB, Neelands TR (2008). Transient receptor potential A1 mediates an osmotically activated ion channel. Eur J Neurosci.

[4] Jordt SE, Bautista DM, Chuang HH, McKemy DD, Zygmunt PM, Hogestatt ED, Meng ID, Julius D (2004). Mustard oils and cannabinoids excite sensory nerve fibres through the TRP channel ANKTM1. Nature.

[5] Kwan KY, Allchorne AJ, Vollrath MA, Christensen AP, Zhang DS, Woolf CJ, Corey DP (2006). TRPA1 contributes to cold, mechanical, and chemical nociception but is not essential for hair-cell transduction. Neuron.

[6] Obata K, Katsura H, Mizushima T, Yamanaka H, Kobayashi K, Dai Y, Fukuoka T, Tokunaga A, Tominaga M, Noguchi K (2005). TRPA1 induced in sensory neurons contributes to cold hyperalgesia after inflammation and nerve injury. J Clin Invest.

[7] Story GM, Peier AM, Reeve AJ, Eid SR, Mosbacher J, Hricik TR, Earley TJ, Hergarden AC, Andersson DA, Hwang SW (2003). ANKTM1, a TRP-like channel expressed in nociceptive neurons, is activated by cold temperatures. Cell.

[8] Bandell M, Story GM, Hwang SW, Viswanath V, Eid SR, Petrus MJ, Earley TJ, Patapoutian A (2004). Noxious cold ion channel TRPA1 is activated by pungent compounds and bradykinin. Neuron.

[9] Petrus M, Peier AM, Bandell M, Hwang SW, Huynh T, Olney N, Jegla T, Patapoutian A (2007). A role of TRPA1 in mechanical hyperalgesia is revealed by pharmacological inhibition. Mol Pain.

[10] Andersson DA, Gentry C, Moss S, Bevan S (2009). Clioquinol and pyrithione activate TRPA1 by increasing intracellular Zn^2+^. Proc Natl Acad Sci USA.

[11] Doerner JF, Gisselmann G, Hatt H, Wetzel CH (2007). Transient receptor potential channel A1 is directly gated by calcium ions. J Biol Chem.

[12] Hu H, Bandell M, Petrus MJ, Zhu MX, Patapoutian A (2009). Zinc activates damage-sensing TRPA1 ion channels. Nat Chem Biol.

[13] Wang YY, Chang RB, Waters HN, McKemy DD, Liman ER (2008). The nociceptor ion channel TRPA1 is potentiated and inactivated by permeating calcium ions. J Biol Chem.

[14] Zurborg S, Yurgionas B, Jira JA, Caspani O, Heppenstall PA (2007). Direct activation of the ion channel TRPA1 by Ca^2+^. Nat Neurosci.

[15] Bautista DM, Movahed P, Hinman A, Axelsson HE, Sterner O, Hogestatt ED, Julius D, Jordt SE, Zygmunt PM (2005). Pungent products from garlic activate the sensory ion channel TRPA1. Proc Natl Acad Sci USA.

[16] Hinman A, Chuang HH, Bautista DM, Julius D (2006). TRP channel activation by reversible covalent modification. Proc Natl Acad Sci USA.

[17] Macpherson LJ, Dubin AE, Evans MJ, Marr F, Schultz PG, Cravatt BF, Patapoutian A (2007). Noxious compounds activate TRPA1 ion channels through covalent modification of cysteines. Nature.

[18] Hu H, Tian J, Zhu Y, Wang C, Xiao R, Herz JM, Wood JD, Zhu MX (2010). Activation of TRPA1 channels by fenamate nonsteroidal anti-inflammatory drugs. Pflugers Arch.

[19] Fujita F, Moriyama T, Higashi T, Shima A, Tominaga M (2007). Methyl p-hydroxybenzoate causes pain sensation through activation of TRPA1 channels. Br J Pharmacol.

[20] Fujita F, Uchida K, Moriyama T, Shima A, Shibasaki K, Inada H, Sokabe T, Tominaga M (2008). Intracellular alkalization causes pain sensation through activation of TRPA1 in mice. J Clin Invest.

[21] Xiao B, Dubin AE, Bursulaya B, Viswanath V, Jegla TJ, Patapoutian A (2008). Identification of transmembrane domain 5 as a critical molecular determinant of menthol sensitivity in mammalian TRPA1 channels. J Neurosci.

[22] Karashima Y, Damann N, Prenen J, Talavera K, Segal A, Voets T, Nilius B (2007). Bimodal action of menthol on the transient receptor potential channel TRPA1. J Neurosci.

[23] Zhou Y, Suzuki Y, Uchida K, Tominaga M (2013) Identification of a splice variant of mouse TRPA1 that regulates TRPA1 activity. Nat Commun 4:2408 10.1038/ncomms3399 PMC5882232 24008932

[24] Bautista DM, Jordt SE, Nikai T, Tsuruda PR, Read AJ, Poblete J, Yamoah EN, Basbaum AI, Julius D (2006). TRPA1 mediates the inflammatory actions of environmental irritants and proalgesic agents. Cell.

[25] Caceres AI, Brackmann M, Elia MD, Bessac BF, del Camino D, D’Amours M, Witek JS, Fanger CM, Chong JA, Hayward NJ (2009). A sensory neuronal ion channel essential for airway inflammation and hyperreactivity in asthma. Proc Natl Acad Sci USA.

[26] Chen J, Joshi SK, DiDomenico S, Perner RJ, Mikusa JP, Gauvin DM, Segreti JA, Han P, Zhang XF, Niforatos W (2011). Selective blockade of TRPA1 channel attenuates pathological pain without altering noxious cold sensation or body temperature regulation. Pain.

[27] Eid SR, Crown ED, Moore EL, Liang HA, Choong KC, Dima S, Henze DA, Kane SA, Urban MO (2008). HC-030031, a TRPA1 selective antagonist, attenuates inflammatory- and neuropathy-induced mechanical hypersensitivity. Mol Pain.

[28] Klionsky L, Tamir R, Gao B, Wang W, Immke DC, Nishimura N, Gavva NR (2007). Species-specific pharmacology of Trichloro(sulfanyl)ethyl benzamides as transient receptor potential ankyrin 1 (TRPA1) antagonists. Mol Pain.

[29] McGaraughty S, Chu KL, Perner RJ, Didomenico S, Kort ME, Kym PR (2010). TRPA1 modulation of spontaneous and mechanically evoked firing of spinal neurons in uninjured, osteoarthritic, and inflamed rats. Mol Pain.

[30] Nagata K, Duggan A, Kumar G, Garcia-Anoveros J (2005). Nociceptor and hair cell transducer properties of TRPA1, a channel for pain and hearing. J Neurosci.

[31] Xu H, Blair NT, Clapham DE (2005). Camphor activates and strongly desensitizes the transient receptor potential vanilloid subtype 1 channel in a vanilloid-independent mechanism. J Neurosci.

[32] Chen J, Zhang XF, Kort ME, Huth JR, Sun C, Miesbauer LJ, Cassar SC, Neelands T, Scott VE, Moreland RB, Reilly RM, Hajduk PJ, Kym PR, Hutchins CW, Faltynek CR (2008). Molecular determinants of species-specific activation or blockade of TRPA1 channels. J Neurosci.

[33] Galeotti N, Ghelardini C, Mannelli L, Mazzanti G, Baghiroli L, Bartolini A (2001). Local anaesthetic activity of (+)- and (−)-menthol. Planta Med.

[34] Galeotti N, Di Cesare Mannelli L, Mazzanti G, Bartolini A, Ghelardini C (2002). Menthol: a natural analgesic compound. Neurosci Lett.

[35] Santos FA, Rao VS (2001). 1,8-cineol, a food flavoring agent, prevents ethanol-induced gastric injury in rats. Dig Dis Sci.

[36] Umezu T, Sakata A, Ito H (2001). Ambulation-promoting effect of peppermint oil and identification of its active constituents. Pharmacol Biochem Behav.

[37] Anand P (2003). Capsaicin and menthol in the treatment of itch and pain: recently cloned receptors provide the key. Gut.

[38] Sherkheli MA, Benecke H, Doerner JF, Kletke O, Vogt-Eisele AK, Gisselmann G, Hatt H (2009). Monoterpenoids induce agonist-specific desensitization of transient receptor potential vanilloid-3 (TRPV3) ion channels. J Pharm Pharm Sci.

[39] Takaishi M, Fujita F, Uchida K, Yamamoto S, Sawada Shimizu M, Hatai Uotsu C, Shimizu M, Tominaga M (2012). 1,8-cineole, a TRPM8 agonist, is a novel natural antagonist of human TRPA1. Mol Pain.

[40] Vogt-Eisele AK, Weber K, Sherkheli MA, Vielhaber G, Panten J, Gisselmann G, Hatt H (2007). Monoterpenoid agonists of TRPV3. Br J Pharmacol.

[41] Vallin KS, Sterky KJ, Nyman E, Bernström J, From R, Linde C, Minidis AB, Nolting A, Närhi K, Santangelo EM, Sehgelmeble FW, Sohn D, Strindlund J, Weigelt D (2012). *N*-1-Alkyl-2-oxo-2-aryl amides as novel antagonists of the TRPA1 receptor. Bioorg Med Chem Lett.

[42] Chen J, Joshi SK, DiDomenico S, Perner RJ, Mikusa JP, Gauvin DM, Segreti JA, Han P, Zhang XF, Niforatos W, Bianchi BR, Baker SJ, Zhong C, Simler GH, McDonald HA, Schmidt RG, McGaraughty SP, Chu KL, Faltynek CR, Kort ME, Reilly RM, Kym PR (2011). Selective blockade of TRPA1 channel attenuates pathological pain without altering noxious cold sensation or body temperature regulation. Pain.

[43] Almeida JR, Souza GR, Silva JC, Saraiva SR, Júnior RG, Quintans Jde S, Barreto Rde S, Bonjardim LR, Cavalcanti SC, Junior LJ (2013). Borneol, a bicyclic monoterpene alcohol, reduces nociceptive behavior and inflammatory response in mice. Sci World J.

[44] Silva-Filho JC, Oliveira NN, Arcanjo DD, Quintans-Júnior LJ, Cavalcanti SC, Santos MR, Oliveira RD, Oliveira AP (2011). Investigation of mechanisms involved in (−)-borneol-induced vasorelaxant response on rat thoracic aorta. Basic Clin Pharmacol Toxicol.

[45] Liu R, Zhang L, Lan X, Li L, Zhang TT, Sun JH, Du GH (2011). Protection by borneol on cortical neurons against oxygen–glucose deprivation/reperfusion: involvement of anti-oxidation and anti-inflammation through nuclear transcription factor κappaB signaling pathway. Neuroscience.

[46] Park TJ, Park YS, Lee TG, Ha H, Kim KT (2003). Inhibition of acetylcholine-mediated effects by borneol. Biochem Pharmacol.

[47] Masakazu Y (1988). Musty odour problems in Lake Biwa 1982-1987. Water Sci Technol.

[48] Miyazawa M, Yamafuji C (2005). Inhibition of acetylcholinesterase activity by bicyclic monoterpenoids. J Agric Food Chem.

[49] Alpizar YA, Gees M, Sanchez A, Apetrei A, Voets T, Nilius B, Talavera K (2013). Bimodal effects of cinnamaldehyde and camphor on mouse TRPA1. Pflugers Arch.

