# Supplementary figures and images for: Inhibitory effects of monoterpenes on human TRPA1 and the structural basis of their activity
Source: J Physiol Sci. 2013 Oct 12;64(1):47–57. doi: 10.1007/s12576-013-0289-0 (PMC3889502; doi:10.1007/s12576-013-0289-0)

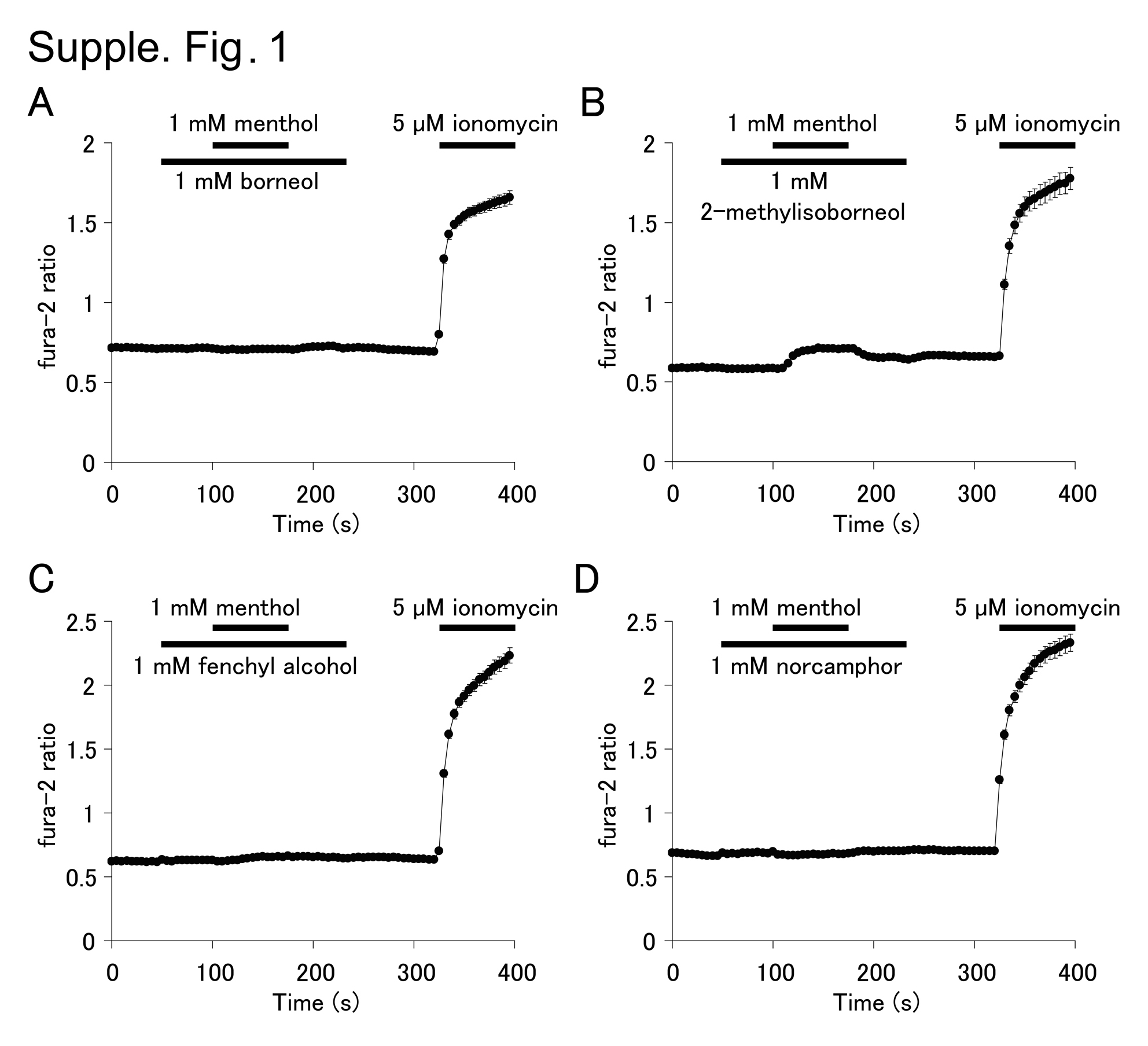

Supplement: Supplementary file 1 — Supplementary material 1 (JPEG 324 kb) Supplementary Figure 1. Effects of borneol, 2-methylisoborneol, fenchyl alcohol and norcamphor with or without menthol on cytosolic Ca 2+ concentrations in vector-transfected HEK293T cells. No changes in the fura-2 ratio were observed while cells responded normally to ionomycin (5 μM). (n = 48-75) [file 12576_2013_289_MOESM1_ESM.jpg]

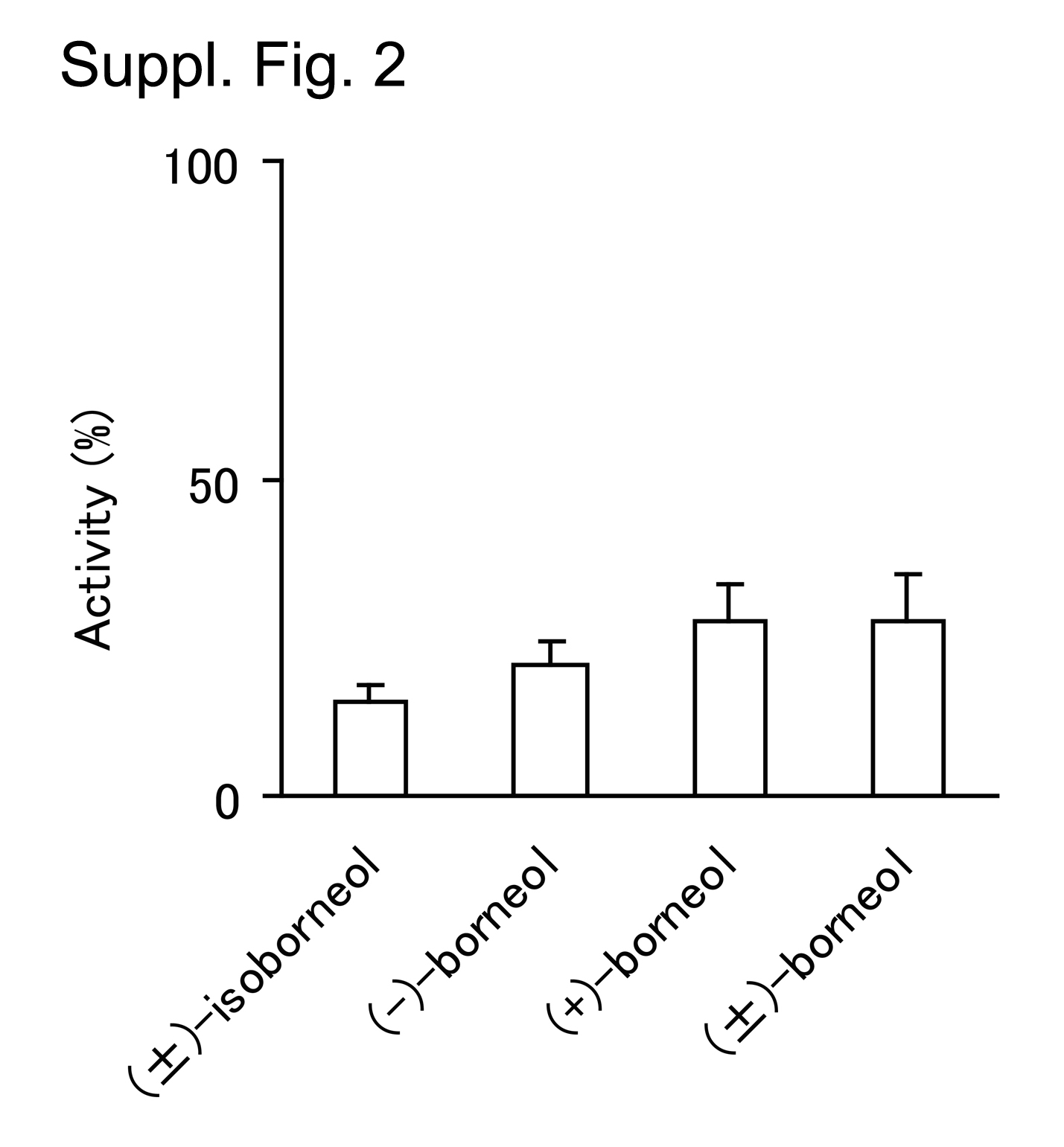

Supplement: Supplementary file 2 — Supplementary material 2 (JPEG 126 kb) Supplementary Figure 2 Comparison of the inhibitory effects on AITC (20 μM)-induced hTRPA1 current among borneol isomers and (±) isoborneol. (n = 5-6) [file 12576_2013_289_MOESM2_ESM.jpg]
